# Dust Mite-Specific IgE in Nasal Lavage Fluid During Natural Allergen Exposure and After Nasal Provocation Test in Subjects with Suspected Local Allergic Rhinitis

**DOI:** 10.3390/life15111683

**Published:** 2025-10-29

**Authors:** Mohamad Mahdi Mortada, Alaa Sherri, Edyta Pietrowska, Marta Popławska, Maciej Chałubiński, Marcin Kurowski

**Affiliations:** 1 Department of Immunology and Allergy, International Doctoral School, Medical University of Łódź, 92-213 Łódź, Poland; mohamadmahdi.mortada1@student.umed.lodz.pl (M.M.M.);; 2 Department of Rheumatology, Medical University of Łódź, 90-549 Łódź, Poland; alaa.sherri@umed.lodz.pl; 3 Biobank HARC, Medical University of Łódź, 92-213 Łódź, Poland

**Keywords:** local allergic rhinitis, specific immunoglobulin E (IgE), nasal mucosa, nasal provocation test, dust mite allergens, nasal lavage

## Abstract

**Introduction:** Apart from the typical AR phenotype and its standard clinical manifestations—rhinorrhea, sneezing, nasal itching, and congestion—the so-called local allergic rhinitis (LAR) can be observed in a subset of subjects presenting rhinitis symptoms, a negative skin prick test (SPT), and serum-specific immunoglobulin E (sIgE) for the relevant allergens and confirmed with a positive nasal provocation test (NPT), which is the gold standard in LAR diagnosis. Our study aims to assess the clinical symptoms and local mucosal sIgE presence induced by NPT and natural exposure to HDM allergens in subjects with suspected LAR. **Methods:** In total, 25 suspected LAR subjects were included in the study. The total nasal symptom score (TNSS) and visual analog scale (VAS) were used for the subjective assessment. A nasal provocation test (NPT) was performed with house dust mite allergens. The nasal lavage technique was used for nasal secretion acquisition, in which the levels of sIgE were measured. **Results**: During the period of increased exposure vs. the off-exposure period, the TNSS and VAS were significantly higher (*p* = 0.0361 and *p* = 0.0031, respectively). Levels of IgE specific to *Dermatophagoides pteronyssinus* in nasal lavage were high (*p* = 0.0502). Similarly, high levels of sIgE to *Dermatophagoides farinae* were noted (*p* = 0.0164). Comparing pre-NPT and post-NPT results, LAR diagnosis was confirmed in 8 subjects. Only the VAS score was higher after a positive NPT. Both sIgE to *Dermatophagoides pteronyssinus* and *Dermatophagoides farinae* in nasal lavage were higher after a positive NPT; however, the change was not statistically significant. A higher fold change in the median relative value (sIgE/Total IgE) for both allergens was noted in the positive-NPT group compared to the negative-NPT group. **Conclusions:** Assessing the local nasal production of sIgE and other inflammatory mediators may contribute to expanding our knowledge of LAR pathogenesis. Further studies including a larger number of subjects are needed for a better understanding of the LAR entity in terms of diagnosis and treatment options.

## 1. Introduction

Allergic rhinitis (AR) is a very common condition affecting both adult and pediatric populations, with a prevalence of 30% and 40%, respectively [1]. Apart from the typical AR phenotype and its standard clinical manifestations—rhinorrhea, sneezing, nasal itching, and congestion—the so-called local allergic rhinitis (LAR), which was first described in 2009 [2], can be observed in a subset of subjects. LAR is suspected in subjects presenting rhinitis symptoms, a negative skin prick test (SPT), and serum-specific immunoglobulin E (sIgE) for the relevant allergens, and confirmed with a positive nasal provocation test (NPT), which is the gold standard in LAR diagnosis [2]. Among subjects with rhinitis symptoms, the prevalence of LAR falls between 21% and 62.5% for the adult population and between 3% and 66.7% for the pediatric population [3,4,5,6]. The noted difference in prevalence depends on the population studied and the methodology employed. LAR patients are usually either underdiagnosed or misdiagnosed and assigned to the non-allergic rhinitis group (NAR) [7]. Additionally, in a subset of subjects with the coexistence of both AR and LAR, dual allergic rhinitis (DAR) can be described [8]. The mechanisms of local mucosal allergic inflammation occurring during LAR have not been fully elucidated; however, several investigations have been carried out regarding the possible pathophysiology of LAR. Basophil activation tests (BATs) performed on subjects with suspected LAR show a positive result in a considerable proportion of cases [9]. The concept of type 2 inflammation evolves not only through the activity of Th2 lymphocytes but also through the production of alarmins at the level of the respiratory epithelium. In addition to sIgE, several mediators and proteins (e.g., IL-25/IL-33/TSLP, IL17, IL-4, IFN-γ, Tryptase/ECP, IL1-ra, TNF-α, LDH, and YKL-40) have been assessed in LAR subjects, and research work in this context has shown promising results [2,10,11,12,13,14,15]. As LAR diagnosis still poses challenges for physicians, the pioneering nature of our research project lies in the quest to unravel the underlying mechanisms of LAR and enhance the diagnostic approach. Ultimately, with the proper identification of the affected individuals, a modified management plan including allergen immunotherapy (AIT) could be implemented in the treatment of LAR subjects. It has been proven that LAR subjects receiving AIT showed a decrease not only in their daily symptoms but also in their risk of developing asthma later in their lives [16]. However, until today, LAR has not been included as an indication for immunotherapy in current guidelines [17]. We hypothesize that in a considerable proportion of patients in whom symptoms are suggestive of seasonal or perennial allergic rhinitis but sIgE cannot be ascertained, an allergic inflammation process and IgE-mediated reaction may occur only at the level of the local mucosa. This study mainly aims to assess the clinical symptoms and local mucosal sIgE presence induced by NPT and natural exposure to house dust mite (HDM) allergens in subjects with suspected LAR. An additional objective of our study was to evaluate whether the nasal provocation test (NPT) can be incorporated into routine clinic visits to assess suspected LAR subjects. For this reason, the relatively short duration of 15 min was selected, as a longer duration might discourage the subjects from participating in the study.

## 2. Population and Methods

### 2.1. Subjects’ Recruitment

Patients included in this study had been referred to the allergy outpatient clinic with suspected allergic rhinitis and underwent a standard diagnostic assessment, including a detailed symptom history and IgE determination through skin prick testing and serum-specific IgE assessment. LAR was suspected based upon the presence of perennial symptoms, under circumstances presumably involving dust mite exposure, negative SPT results, and the absence of specific IgE to *Dermatophagoides pteronyssinus* and *Dermatophagoides farinae* and other perennial allergens in serum. Patients with a history of chronic rhinosinusitis, nasal polyposis, significant nasal septum deviation, and other laryngological conditions that could interfere with the possible outcomes of the investigation were excluded from the study. Asthmatic patients were not excluded a priori. Spirometry was performed before provocation to identify subjects with decreased lung function. None of the included subjects had FEV1 < 80%; therefore, there was no need to postpone NPT or exclude any participant on that basis. Three of the recruited subjects had had a history of mild asthma, for which treatment had commenced in the past with inhaled glucocorticosteroids at a dose of 200 μg budesonide or equivalent, and this was pursued at a stable dose when they presented to the clinic for evaluation of their rhinitis symptoms. Their symptoms remained well-controlled, with no exacerbation reported in the past 12 months, and no adjustments to the treatment regimen were made.

Subjects were recruited between the period of June 2022 and the end of July 2023 in the outpatient clinic of the Department of Immunology and Allergy at the Medical University of Lodz, Poland. All recruited subjects received detailed explanations of the methods used in the sample collection and signed written consent for participation in the study. The study was approved by the Bioethics Committee of the Medical University of Lodz (decision no. RNN/76/22/KE dated 10 May 2022).

### 2.2. Skin Prick Tests (SPTs) and Serum IgE Assessment

Skin prick tests were performed using a panel of airborne allergens, including *Dermatophagoides pteronyssinus*, *Dermatophagoides farinae*, alder, hazel, birch, grass pollen mix, rye, mugwort, cat, dog, *Alternaria*, and *Cladosporium* (Diater, Leganés, Spain). The wheal and flare sizes were measured after 15 min, and wheals with a diameter ≥3 mm were considered positive. Histamine hydrochloride (1 mg/mL) and buffer solution served as controls for positive and negative reactions, respectively. Serum-specific IgE levels to airborne allergens were assessed using EUROLINE allergy diagnostic profiles (EUROIMMUN Medizinische Labordiagnostika AG, Lübeck, Germany). Additionally, the sIgE levels of the *Dermatophagoides pteronyssinus* and *Dermatophagoides farinae* allergen extracts were assessed using an ImmunoCAP immunoassay (Thermo Fisher Scientific, Uppsala, Sweden). Serum-specific IgE concentrations exceeding the threshold of 0.35 kUA/L were considered positive, as indicated by the manufacturers of the diagnostic tools.

### 2.3. Total Nasal Symptom Score (TNSS) and Visual Analog Scale (VAS)

TNSS is the sum of scores for nasal congestion, sneezing, nasal itching, and rhinorrhea using a four-point scale (0–3), where 0 indicates no symptoms, a score of 1 indicates mild symptoms, 2 indicates symptoms that are bothersome but tolerated by the subject, and 3 indicates severe symptoms. TNSS was calculated by adding the scores for each of the symptoms to a total of 12. The VAS is a 100 mm scale that ranges from “no symptoms” to “worst symptoms ever”, applied to assess the overall intensity of the rhinitis symptoms [18].

### 2.4. Nasal Provocation Test (NPT)

NPT was performed outside of the exposure period to HDM allergens using dissolved immunotherapy tablets (ACARIZAX, ALK, Copenhagen, Denmark) according to the protocol published by Horn [19]. NPT was applied by spraying a low concentration of the solution (0.15 mL-puff of 0.014 SQ-HDM) and, if negative, a higher concentration (0.36 SQ-HDM) into the same nostril. Fifteen minutes after the provocation, the following measures were performed: (1) The subjects completed the TNSS/VAS questionnaires (also completed before NPT). (2) The subjects were assessed by a physician through rhinoscopy for any of the following symptoms: swelling, redness, or discharge. (3) Nasal secretions were collected through the nasal lavage method. The NPT results were interpreted based on the EAACI position paper on the standardization of nasal allergen challenges as well as the work group report of the American Academy of Allergy, Asthma, and Immunology [20,21], and the increase in TNSS and VAS scores was assessed, without using the tools such as peak nasal inspiratory flow (PNIF), acoustic rhinometry (AcRh), active anterior rhinomanometry (AAR), or 4-phase rhinomanometry (4PR). The interpretation criteria were as follows:

Group A (negative NPT): No symptoms on objective assessment through anterior rhinoscopy + no increase in either TNSS or VAS scores.

Group B (positive NPT): Presence of symptoms on objective assessment through anterior rhinoscopy + mild/moderate increase in either TNSS or VAS scores.

### 2.5. Nasal Secretion Acquisition

The nasal secretions were acquired through the nasal lavage technique, which is a noninvasive technique that allows for the collection of samples in a short period, as described previously [22,23]. In the nasal lavage technique, the subject is asked to sit on a chair and lean forward; then, a volume of 5 mL of saline solution is administered to one nostril through a syringe connected to an olive, and after 10 min, the solution is retrieved, and the collected volume is noted. Nasal secretions were acquired before and after NPT, as well as during the season of increased exposure to HDM (fall–winter seasons in Poland, usually in an environment with low temperature and increased indoor humidity due to internal heating) and during the off-exposure period (pre-NPT measurement). Nasal lavage samples were centrifuged at 3000 rpm at 4 °C for 15 min and stored at −70 °C until analysis. The dilution of nasal secretion by lavage fluid was estimated according to the previously published formula [24] as follows: dilution factor (DF) = [urea serum] 1.2/[urea in NLF]. Then, we calculated the sIgE concentration in the collected nasal secretion by multiplying DF by NLF concentration. Urea levels in serum and nasal secretions were measured spectrophotometrically using a commercial kit (Cell Biolabs, Inc., San Diego, CA, USA).

### 2.6. Local Specific Immunoglobulin E (sIgE) and Total IgE

sIgE to HDM allergens (D1, *Dermatophagoides pteronyssinus*; D2, *Dermatophagoides farinae*) in nasal secretions, obtained as described previously, was measured using an ImmunoCAP immunoassay (Thermo Fischer Scientific, Uppsala, Sweden). Total IgE in nasal secretions was measured using the ImmunoCap immunoassay (Thermo Fischer Scientific, Uppsala, Sweden).

### 2.7. Statistical Analysis

Statistical analysis was performed using GraphPad Prism version 10.1.2 for Windows (GraphPad Software, Boston, MA, USA). The normality of the data distribution was tested using the Shapiro–Wilk test. The nonparametric Mann–Whitney test and Wilcoxon matched-pairs signed-rank test were used to evaluate the significance of the differences between the compared groups. The differences were considered significant at a *p*-value of <0.05.

## 3. Results

### 3.1. HDM Exposure Period vs. Off-Exposure Period

A total of 25 suspected LAR subjects were included in the study (Table 1). The median age of female subjects was 46 years, with a range of 22–71 years, while for male subjects, the median age was 26.5, with a range of 18–57 years. Due to the inequality of the number of samples collected from the subjects during the exposure period compared to samples collected outside of the exposure period, raw data (without DF correction) were used for the analysis of those groups. As paired comparisons were possible in the assessment before and after provocation, data corrections according to the DF were implemented. Both TNSS and VAS scores were significantly higher during the period of increased exposure to HDM allergens compared to the off-exposure period (Table 2). During the period of increased exposure, specific IgE to *Dermatophagoides pteronyssinus* measured in nasal lavage was significantly higher compared to its level in the off-exposure period. Similarly, higher levels were noted for sIgE to *Dermatophagoides farinae*, but the difference was not significant (Table 3).

### 3.2. Pre-NPT vs. Post-NPT

Out of the 25 NPTs performed, 8 were positive, confirming the diagnosis of LAR in those subjects (Table 3). The VAS score was significantly higher in Group B (median = 58.5 +/− [48.75–72]) compared to the pre-NPT result (median = 27.5 +/− [22.5–65.75]). Surprisingly, the TNSS was lower (Table 3). In Group A, lower median TNSS and VAS scores were noted after nasal provocation (Table 3). 

**Figure 1:**
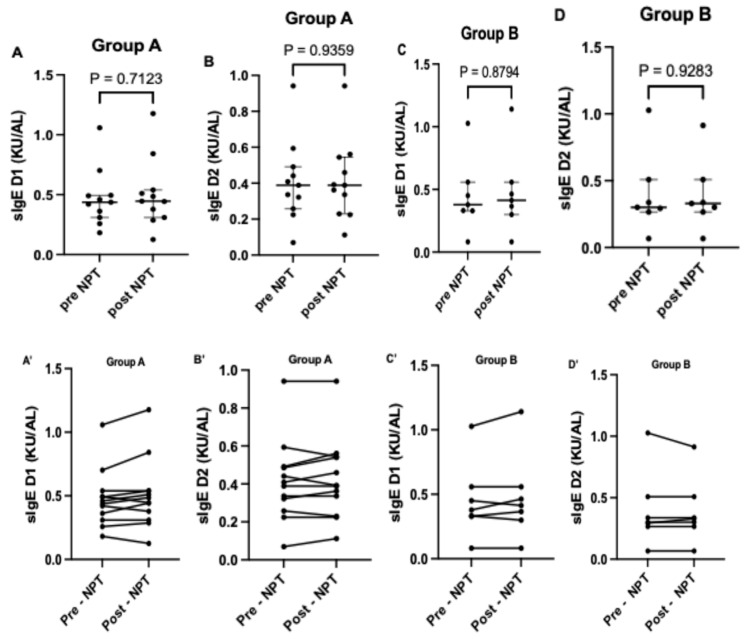
Comparison of sIgE to D1 and D2 pre- and post-provocation in Groups A and B. Figures (**A**–**D**) shows the median level of sIgE to D1 and D2 pre and post nasal provocation for groups A and B respectively. (**A’**–**D’**) shows the individual level of sIgE to D1 and D2 pre and post nasal provocation for each subjects in groups A and B respectively. ***sIgE***, specific immunoglobulin E; ***D1***, *Dermatophagoides pteronyssinus*; ***D2***, *Dermatophagoides farinae*.

In Group A, a higher level of sIgE to *Dermatophagoides pteronyssinus* was noted post-NPT compared to the pre-NPT assessment (Figure 1A), while for sIgE to *Dermatophagoides farinae*, the levels were similar (Figure 1B). In Group B, the sIgE levels to both allergens, *Dermatophagoides pteronyssinus* and *Dermatophagoides farinae*, were higher post-NPT compared to pre-NPT assessment (Figure 1C,D). Individual changes in sIgE to *Dermatophagoides pteronyssinus* and *Dermatophagoides farinae* before and after provocation in both Groups A and B are demonstrated in Figure 1A’–D’. Median levels of sIgE to both allergens in Groups A and B are presented in Table 4.

In both Groups A and B, and with regard to specific IgE to both dust mites (*Dermatophagoides pteronyssinus* and *Dermatophagoides farinae*), considerable variability in nasal lavage sIgE levels was noted. That variability was observed in the case of pre- as well as post-NPT measurements, and the following values of the coefficient of variation (CV) were ascertained:

- For sIgE to Dermatophagoides pteronyssinus (D1): 50.5% (Group A, pre-NPT); 56.6% (Group A, post-NPT); 64.8% (Group B, pre-NPT); 69.5% (Group B, post-NPT)
- For sIgE to Dermatophagoides farinae (D2): 54.8% (Group A, pre-NPT); 53.3% (Group A, pot-NPT); 76.4% (Group B, pre-NPT); 68.2% (Group B, post-NPT)

### 3.3. Total and Relative Change of sIgE to D1 and D2

**Figure 2:**
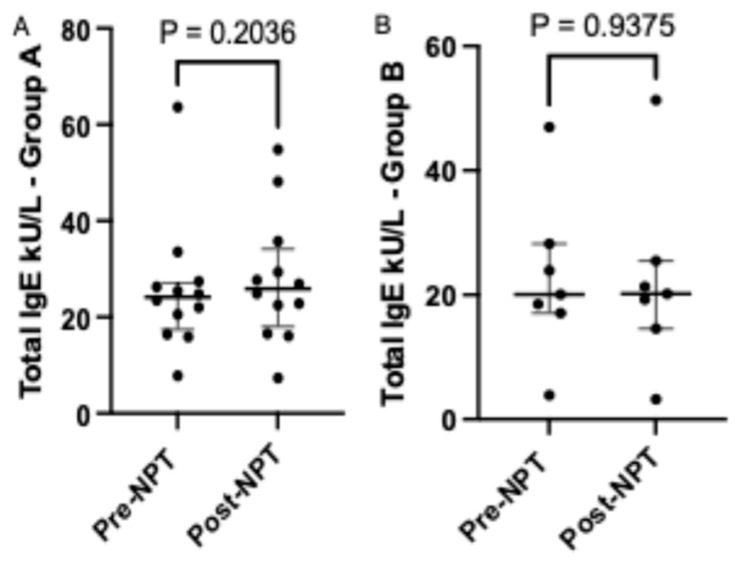
Comparison of total IgE level before and after provocation in Group A and Group B. ***NPT***, nasal provocation test; ***IgE***, immunoglobulin E. Results are presented as median + IQR (interquartile range).

A higher median level of total IgE was noted in nasal lavage from Group A subjects (Figure 2A) after NPT (25.89; [18.06–34.14]) compared to pre-NPT (24.16; [17.45–27.14]). A lower increase in total IgE levels was noted in nasal lavage secretions acquired from Group B (Figure 2B) subjects (20.20; [14.56–25.48]) post-NPT compared to (20.07; [17.09–28.22]) pre-NPT.

**Figure 3:**
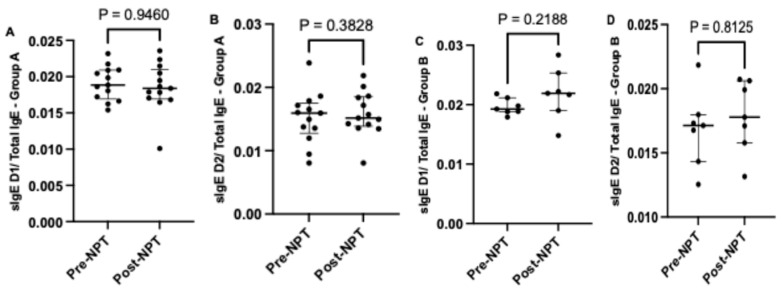
Comparison of relative sIgE levels to both allergens D1 and D2 calculated as sIgE/Total IgE before and after provocation in Group A (**A**,**B**) and Group B (**C**,**D**). ***NPT***, nasal provocation test; ***sIgE***, specific immunoglobulin E; **D1**, *Dermatophagoides pteronyssinus*; **D2**, *Dermatophagoides farinae*. Results are presented as median + IQR (interquartile range).

When comparing the relative sIgE level in lavage (sIgE /Total IgE), we observed a higher fold change in the median relative value for both allergens in the positive-NPT group compared to the negative-NPT group (Figure 3A–D). In the positive-NPT group, a higher relative change in sIgE to *Dermatophagoides pteronyssinus* was noted post-NPT (0.0219; [0.0190–0.0253]) compared to pre-NPT (0.0193; [0.0188–0.0212]). Similarly, the relative change in sIgE to *Dermatophagoides farinae* was higher post-NPT (0.0178; [0.0158–0.0206]) compared to pre-NPT (0.0171; [0.0143–0.0180]).

**Figure 4:**
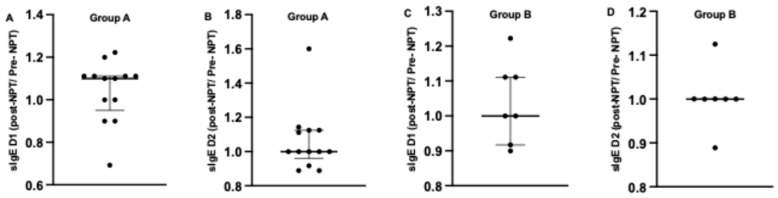
Comparison of individual relative sIgE change calculated as sIgE post-NPT/sIgE pre-NPT to both allergens D1 and D2 before and after provocation in Group A (**A**,**B**) and Group B (**C**,**D**). ***NPT***, nasal provocation test; ***sIgE***, specific immunoglobulin E; **D1**, *Dermatophagoides pteronyssinus*; **D2**, *Dermatophagoides farinae*. Results are presented as median + IQR (interquartile range).

After comparing the individual relative change (sIgE post-NPT/sIgE pre-NPT) (Figure 4), we found the following results: In Group A, a higher median relative change for sIgE to *Dermatophagoides pteronyssinus* (1.1; [0.95–1.111]) was noted post-NPT compared to sIgE to *Dermatophagoides farinae* (1; 90.9583–1.1250]. Furthermore, eight subjects showed a fold change higher than 1.1 for sIgE to *Dermatophagoides pteronyssinus*. For *Dermatophagoides farinae*, five subjects showed a fold change of 1.1 or higher, with 1 subject having a fold change of 1.6.

In Group B, a similar median relative change was noted for sIgE to both allergens: 1; [0.9167–1.111]) and 1; [1-1] for sIgE to *Dermatophagoides pteronyssinus* and *Dermatophagoides farinae*, respectively. Furthermore, more subjects showed an increase in sIgE to *Dermatophagoides pteronyssinus* rather than sIgE to *Dermatophagoides farinae* among the subjects of Group B (Figure 4).

## 4. Discussion

The approach to the diagnostic and management procedures of LAR patients is still considered a challenge for physicians, and a considerable percentage of LAR subjects are still diagnosed as NAR, as mentioned previously. Given the double-negative results seen in LAR subjects, neither SPT nor serum-specific IgE measurement is of value in the diagnostic assessment. Currently, in LAR diagnosis, the NPT is considered the gold standard [9]; however, this method is time-consuming and requires experienced personnel to be performed correctly.

In our study, LAR was confirmed through NPT in 34% of the subjects. Concerning the subjective assessment, only the VAS score was higher after a positive NPT. Although TNSS/VAS are routinely used tools in subjective assessment, discrepancies in results may arise, as was the case in our study. For example, we noted that post-NPT, several subjects showed local mucosal symptoms such as redness, swelling, as well as nasal discharge when assessed through anterior rhinoscopy (physician assessment); however, surprisingly, when they completed the questionnaire, a lower TNSS result was noted among them. Thus, relying on TNSS/VAS scores alone could impact the final assessment of the post-NPT manifestations in the subjects.

After a negative NPT, lower scores for both TNSS and VAS were noted. Specific IgE to HDM allergens measured in nasal lavage was higher after a positive NPT. One study showed that in LAR patients, a positive NPT to *Dermatophagoides pteronyssinus* is associated with a significant increase in sIgE and inflammatory mediators detected in nasal secretions [25]. Similarly, in another study performed on 219 subjects, 46 were diagnosed with LAR (29 of them were sensitized to *Dermatophagoides pteronyssinus*), and it was proved that the expression of clinical symptoms after the nasal challenge is associated with a significant increase in nasal sIgE production [4]. Thus, a correlation between nasal sIgE production and the occurrence of symptoms was noted among LAR patients. In our study, we noted that during the period of increased HDM exposure, sIgE to *Dermatophagoides pteronyssinus* in nasal lavage secretions was higher compared to the off-exposure period. Similarly, a significantly higher level was noted for sIgE to *Dermatophagoides farinae*. The nasal secretions from subjects with negative NPT were also assessed for specific IgE to both allergens. Surprisingly, sIgE to *Dermatophagoides pteronyssinus* was higher after a negative NPT compared to pre-NPT, while for *Dermatophagoides farinae*, sIgE levels after a negative NPT were similar.

A similar study including 60 AR subjects also showed that high levels of sIgE to *Dermatophagoides farinae* as well as inflammatory mediators (eosinophil cationic protein (ECP), interleukin 8 (IL-8), and vascular endothelial growth factor (VEGF)) could be detected in nasal secretions after a negative NPT. Moreover, correlations were established between sIgE levels and levels of the inflammatory mediators, suggesting a possible role of locally synthesized sIgE in the mucosal inflammatory process in AR subjects [26]. In another study analyzing the diagnostic utility of assessing sIgE in nasal secretions in different types of rhinitis [27], it was demonstrated that the median level of sIgE to *Dermatophagoides pteronyssinus* in rhinitis patients with atopy was significantly higher than in non-atopic rhinitis subjects. Also, the median level of sIgE in the group of rhinitis patients without atopy was relatively low, yet still detectable.

Comparable low levels of sIgE to both allergens *Dermatophagoides pteronyssinus* and *Dermatophagoides farinae* in nasal secretions were also noted in our study, and several reasons could be behind this finding. One of the main reasons could be the relatively short time between provocation and the acquisition of nasal secretions, which was 15 min in our analysis. In one study, a low sIgE level to *Dermatophagoides pteronyssinus* was detected 15 min after nasal provocation; however, after 1 h, 6 h, and 24 h, a gradually higher level was noted [25]. In another study where LAR was confirmed in 21 out of 84 NAR recruited subjects, the assessment of sIgE to *Dermatophagoides pteronyssinus* in nasal secretions in the LAR subjects showed an increase of sIgE level at 1 h post-NPT compared to 15 min post-provocation, followed by a slight decrease 6 h post-NPT [28]. In the same study, a similar pattern of sIgE levels was noted after provocation with *Alternaria*, but with a higher decrease at 6 h post-NPT. Similar results have also been demonstrated in another study assessing sIgE levels to timothy grass, birch, and mugwort in nasal secretions of LAR subjects [29]. Thus, a prolongation of the time between NPT and nasal secretion acquisition could help increase the amount of sIgE at the assessment time. In our study, a relatively minimal increase in total IgE was noted after positive as well as negative NPT. In our study, a higher median relative change for sIgE to *Dermatophagoides pteronyssinus* was noted post-NPT compared to sIgE to *Dermatophagoides farinae* after a negative NPT. After a positive NPT, a similar median relative change was noted for sIgE to both allergens.

One reason for the low sIgE level could be the decrease in sensitivity due to the dilution of the collected sample through the nasal lavage technique. Therefore, the usefulness of the methods used for nasal secretion acquisition should be assessed, and this was one of the aims of our study. As an implication of this, we decided to perform corrections for the possible dilution. Various methods have been employed to adjust mediator and antibody concentrations for the dilution of nasal secretions in previously performed studies. These included, among others, the calculation of the dilution factor based on a comparison of the concentrations of urea or total protein in serum versus nasal lavage fluid [24,28]. In other studies of a similar design, however, correction for a possible nasal secretion dilution has not been mentioned [6,29,30].

The concentration of specific IgE in nasal secretion, which could be considered a cutoff value for a positive NPT, has also been debated [31]. The cutoff values used vary between studies, and concentrations of 0.1 kUA/L [11], 0.12 kUA/L [27], 0.145 kUA/L [32], and 0.35 kUA/L [25,32] have been used. Cutoff values were chosen based on their calculated sensitivity and specificity [27,33] or based on the detection method and detection limits. In our analysis, we referred to the level of 0.1 kUA/L as the one indicated by the manufacturer. However, the significance and utility of any threshold value may be influenced by the technique through which nasal secretions are acquired [33].

In addition to the above-listed aspects, the considerable variability of sIgE levels should be noted and taken into consideration while implementing and interpreting the results of the measurements. In particular, implications for allergy practice—if considered—should be set forth and delineated with caution. In our sample, the variability of sIgE measurements (as reflected by the CV values) may be considered high. This observation can result from the inter-subject differences and variations characterizing local mucosal reaction to allergen stimulation. Moreover, the considerable variability we have observed further warrants the need for the development and uniformization not only of the tools and procedures for nasal lavage sampling but also of the applied corrections for sample dilution.

Ultimately, the evaluation of mediators as well as sIgE in nasal secretions can provide a better assessment of the allergic and inflammatory processes occurring in the nasal mucosa in LAR subjects. This can hasten the process of diagnosis and thus allow those patients to benefit from the available treatment plans earlier. It is worth mentioning here that delayed diagnosis not only led to increased suffering of the patients due to symptoms interfering with their daily activities, but was also associated with a higher risk of asthma development in the affected subjects. This finding was emphasized in one of the follow-up studies, where 171 LAR patients were assessed continuously for 10 years and were found to have an increase in rhinitis symptoms and an associated need for medical assistance [16]. In the context of the possible occurrence of highly pronounced nasal symptoms and the frequent coexistence of LAR with asthma [34], one of the main positive outcomes of early LAR diagnosis and treatment could be the prevention of asthma. Hence, a better diagnostic approach can allow LAR patients to benefit from immunotherapy, which has been proven not only to reduce symptoms successfully but also to decrease the need for continuous symptomatic treatment [35].

In comparison with previously published studies tackling the same topic, our study is focused on the diagnostic workup for patients with suspected LAR, thus confirming the diagnosis of this entity as well as assessing the variation in clinical symptoms and sIgE levels between the exposure and off-exposure periods as well as post-provocation. In other words, as we considered our study to be pilot in nature, we focused on comparing individual changes in sIgE levels as well as variations in subjective symptoms, assessed through TNSS and VAS questionnaire, and objective symptoms (swelling, redness, or discharge), assessed through anterior rhinoscopy, among the subjects themselves rather than performing an intergroup comparison (with AR or DAR subjects), as noted in similar studies on LAR.

### Limitations

Although in our study we were able to show that an increase in sIgE levels to HDM can be noted after nasal provocation as well as during the exposure season, the changes were not significant. Based on the nature of our study, this lack of significance was foreseen, mainly due to the small size of the included population, which results firstly in higher variability, less precise estimates, as well as limited generalizability, thereby reducing the ability to detect the true effects of nasal provocation and natural HDM exposure on LAR subjects. A second limitation of our study was the exclusion of objective assessment tools such as peak nasal inspiratory flow (PNIF), acoustic rhinometry (AcRh), active anterior rhinomanometry (AAR), and 4-phase-rhinomanometry (4PR). A third limitation in our study, in addition to the small sample size, was the short time (15 min) between provocation and the collection of nasal secretions, which might have led to the relatively low sIgE levels measured in the collected nasal secretion samples, ultimately affecting the statistical comparisons. As mentioned earlier, this 15 min time interval was chosen to assess the feasibility of using it as an assessment tool during a routine clinic visit of suspected LAR subjects, as a longer duration might deter patients from participating in the study. However, our results showed that a possibly longer duration might increase the concentration of the assessed sIgE in lavage secretions. It is also worth mentioning that due to the relatively small size of the study population, including subjects receiving immunotherapy was not a feasible option in our study.

## 5. Conclusions

The possibility of assessing the local production of sIgE and other inflammatory mediators would contribute to expanding knowledge on LAR pathogeneses and provide new insights into phenotypical and endotypical classification of allergic rhinitis. In a small sample-sized study like ours, the clinical relevance could not be effectively assessed. Thus, further studies including a larger number of subjects are needed for a better understanding of the LAR entity in terms of diagnosis and treatment options. A future goal in this context is to work on calculating the threshold value for sIgE in nasal lavage secretions. To achieve this, a well-defined dilution factor protocol and standardized assessment tools for each studied allergen must be implemented separately.

## Figures and Tables

**Table 1:** Descriptive data of the subjects.

Sex	Median Age + Range
**Female (n = 17)**	46 [22–71]
**Male (n = 8)**	31.5 [18–57]

**Table 2:** Comparison of TNSS, VAS, and sIgE to D1 and D2 and total IgE between the exposure period and off-exposure period to HDM allergens.

	Exposure Period	Outside of the Exposure Period	*p*-Value
***TNSS*** ***[0–12]***	6; [3.5–8.5]	3; [1–6]	*p* = 0.0361
***VAS*** ***[0–100]***	61; [52–77]	26; [16–63]	*p* = 0.0031
***sIgE–D1 *** **(kUA/L)**	0.11; [0.1–0.125]	0.1; [0.09–0.11]	*p* = 0.0502
***sIgE–D2 *** **(kUA/L)**	0.1; [0.08–0.1]	0.08; [0.08–0.09]	*p* = 0.0164
***Total IgE *** **(kU/L)**	5.075; [4.878–5.733]	5.370; [4.780–5.610]	*p* = 0.7631

**TNSS**, total nasal symptom score; **VAS**, visual analog scale; **sIgE**, specific immunoglobulin E; **D1**, *Dermatophagoides pteronyssinus*; **D2**, *Dermatophagoides farinae*; Results are presented as median + IQR (interquartile range). In this table, raw data are presented.

**Table 3:** TNSS and VAS scores before and after NPT in Groups A and B.

	TNSS (0–12)		VAS (0–100)	
	Pre-NPT	Post-NPT	Pre-NPT	Post-NPT
**Group A** **(n = 15)**	2; [1–6]	1; [0–2]	24; [5–63]	18; [0.5–31]
**Group B** **(n = 8)**	5; [2.25–5]	3.5; [2.25–5.75]	27.5; [22.5–65.75]	58.5; [48.75–72]
***p* value** **(A vs. B)**	*p* = 0.2026	***p* = 0.0011**	*p* = 0.2579	***p* = 0.0030**

**NPT**, nasal provocation test; **TNSS**, total nasal symptom score; **VAS**, visual analog scale. Results are presented as median + IQR (interquartile range).

**Table 4:** Comparison of sIgE to D1 and D2 in nasal lavage secretions before and after nasal provocation.

	SIgE D1 (kUA/L)		sIgE D2 (kUA/L)	
	Pre NPT	Post NPT	Pre NPT	Post NPT
**Group A, n = 11**	0.4374; [0.3091–0.4948]	0.4454; [0.3091–0.5394]	0.3888; [0.2583–0.4915]	0.3888; [0.2296–0.5443]
**Group B, n = 7**	0.3787; [0.3294–0.5583]	0.4126; [0.2990–0.5583]	0.3001; [0.2658–0.5075]	0.3294; [0.2658–0.5075]
***p* value** **(A vs. B)**	*p* = 0.6590	*p* = 0.5962	*p* = 0.5962	*p* = 0.4525

***NPT***, nasal provocation test; ***sIgE***, specific immunoglobulin E; **D1**, *Dermatophagoides pteronyssinus*; **D2**, *Dermatophagoides farinae*. Results are presented as median + IQR (interquartile range).

## Data Availability

The original contributions presented in this study are included in the article. Further inquiries can be directed to the corresponding author.
